# Book Reviews

**Published:** 1799-05

**Authors:** 


### PRACTICE OF MEDICINE AND SURGERY.

*Medicinæ Praxeos Compendium: ſymptomata, cauſas, diagnoſin, prognoſin, et medendi rationem exhibens.* Auctore, EDVARDO GOODMAN CLARKE, *M. D.* Collegii Regalis Medicorum Londinenſis, nec non Exercitûs Medico. *Londini.* In ædibus auctoris et apud bibliopolarum tabernas habendum. 1799. 214. pp. 12°.—(Price Five Shillings ſewed.)

In a ſhort preface the author informs us, that he has compoſed this abridgment with the greateſt attention, and arranged it chiefly according to the methodical noſology of Cullen. Indeed, it may well ſerve as a ſafe guide to the ſtudent, and occaſionally alſo to the young practitioner; as it affords a conciſe and perſpicuous view of the principal diſeaſes, together with the general indications of cure.

*Medical Strictures; being a conciſe and effectual method of curing the following Diſeaſes: Colds, Ague, Small-pox, Meaſles &c. &c.* By RICHARD CLARKE, M. D. 8vo. *London.* Rider. (Price One Shilling.)

This contemptible publication would deſerve no notice, in our Journal, were it not for the ſake of warning the reader againſt every ſpecies of deluſion which is practiſed by ignorant pretenders, under the ſhield of ſcientific ſemblance. The whole of theſe '*Strictures*' is the effuſion of quackery; for under the pretence of treating and deſcribing about *twenty-three* of the moſt common diſorders, the induſtrious author renders his pamphlet the vehicle of advertiſing *thirty-one* different quack medicines—and *theſe,* according to the title page, are "*the means of prevention, palliation, and cure, diſtinctly pointed out; and the whole adapted to general comprehenſion!*"

*Reflections on the propriety of performing the Cæſarean Operation: to which are added Obſervations on Cancer; and experiments on the ſuppoſed origin of the*

*the Cow-pox.* By W. Simmons, Member of the Corporation of Surgeons in London, and Senior Surgeon to the Manchester Infirmary. *London.* Vernor and Hood.

In this interesting treatise, the author first premises a concise but satisfactory historical account of the Cæsarean operation, and after duly balancing the arguments for and against that fatal section, which has been performed *eleven times* in this kingdom, and has proved mortal in *every instance* *, he concludes this part of his ingenious 'Reflections' with the following decisive opinion on the subject:

"Upon the whole, then, "says Mr. Simmons," in that supposed case of distortion (which I hope will never happen) in which the mother must be doomed to death, from the impossibility of delivering the child by the crotchet, the compound operation I have recommended will furnish a recourse, approved by reason and sanctioned by experience; inasmuch as the section of the symphysis pubis has been made, and the crotchet has been used, though separately, yet with safety. Such a case will be attended, unquestionably, with additional hazard; but it offers the only chance to the mother, to the preservation of whose life, our chief care should be directed: and, I hope, that in future *all trace of the cæsarean operation will be banished from professional books*; for it can never be justifiable during the parents life, and stands recorded only to disgrace the art."

In his practical '*Observations on Cancer*,' Mr. Simmons informs us, that he has prescribed a solution of arsenic, with singular advantage, in a case of confirmed cancer, by directing fifteen drops to be taken three times a day; leaving it off for a few days, on account of an alarming general indisposition; and, when these threatening symptoms had subsided, returning to the former dose.

The author concludes with an account of some *experiments on the supposed origin of the cow-pox*; the result of these we extract in the following passages: "The limitation of the contagious power of the fluid supposed to occasion the cow-pox, and obtained from the horse's heel, to the first or erysipelatous stage of the grease, disproves the identity; and also destroys any analogy, that might have been conceived to subsist between it and variolous matter.

"Twelve punctures were made in the teats of three cows, inoculated with the *ichorous* fluid, and it did not produce the smallest effect in either of them: six children were inoculated with the same sort of fluid, by making four punctures in the left arm of each; and no disease whatever ensued; eight punctures were made in the teats of two cows, and variolous matter was inserted; but not the smallest change took place: one single puncture, with diluted variolous matter, gave the small-pox to a child.

"The evidence, threfore, is as *one* to *twenty four*, in the human subject, between variolous matter, and the discharge from the horse's heel; as *one* to *twelve* in cows: and between the insertion of variolous matter in man, and in cows, as *one* to *eight*.

"These experiments prove, first; that the cow-pox poison does not originate in the horse's heel; secondly, that cows will not take the small-pox."

*Manuel de la saignée, &c. An Epitome of the Practice of Phlebotomy; or, a Dialogue on that Operation.*—By Roch Tarbes. 12mo. (2 livres,) *Paris.* Crullebois.

This

* Osborne's Essays on Midwifery; p. 440.—Since the publication of this work, several other cases have occured, in which the Cæsarean operation is said to have been attended with the same fatal event: vide Mr. Simmons's Reflections. p. 28

This little volume comprifes the moft ufeful information on the fubject here treated; it is written in a manner lively and perfpicuous, fo that it cannot fail to be of fome utility, efpecially to the young practitioner.

