# Errata

**Published:** 1799-06

**Authors:** 


					P* 3?9? '? *4? rorrecourse 9 rearesource.

				

